# Uncuffed Endotracheal Tube Experience in Pediatric Patients with Laparotomy and Laparoscopic Surgeries

**DOI:** 10.1155/2020/6325293

**Published:** 2020-05-09

**Authors:** Sema Şanal Baş, Gülay Erdoğan Kayhan, Meryem Onay, Yeliz Kılıç

**Affiliations:** Department of Anesthesiology and Reanimation, Eskisehir Osmangazi University Faculty of Medicine, Eskisehir, Turkey

## Abstract

**Aim:**

The aim of this study is to compare endotracheal tube leak, tube selection, mechanical ventilation, and side effects in the use of uncuffed tubes in both laparoscopic and laparotomy surgeries in pediatric patients. *Material and Method*. Patients who underwent laparotomy (LT group) or laparoscopic (LS group) surgery between 1 and 60 months. In the selection of uncuffed tubes, it was also planned to start endotracheal intubation with the largest uncuffed tube and to start intubation with a small uncuffed tube if the tube encounters resistance and does not pass. Mechanical parameters, endotracheal tube size, tube changes, and side effects are recorded.

**Results:**

A total of 102 patients, 38 females and 64 males, with a mean age of 10.9 ± 8.1 months, body weight 7.1 ± 3.7 kg, and height 67 ± 15 cm, were included. 54 patients underwent laparoscopic surgery, and 48 patients underwent laparotomy. Tube exchange was performed in a total of 18 patients. In patients who underwent tube exchange, 11 patients were intubated with a smaller ETT number and others endotracheal intubation; when the MV parameters were TVe < 8 ml/kg and ETT leak > 20%, a larger uncuffed tube was used due to PIP 30 cmH_2_O pressure. Patients with aspiration were not found in the LT and LS groups. There was no difference in the intergroup evaluation for postoperative side effects such as cough, laryngospasm, stridor, and aspiration.

**Conclusion:**

There was no significant difference between the groups in terms of tube changes and side effects. So that we can start with the largest possible uncuffed tube to decrease ETT leak, both laparotomy and laparoscopic operations in children can be achieved with safe mechanical ventilation and target tidal volume.

## 1. Introduction

There is a widespread opinion that an uncuffed endotracheal tube (ETT) should be selected as the intubation tube in pediatric patients [1–4]. Despite disadvantages such as air leakage, environmental contamination of anesthetic gases, and aspiration, the application of an uncuffed ETT prevents trauma to the subglottic region in childhood; moreover, a lower airway is often preferred for many reasons, such as the application of resistance [1, 2, 4, 5].

In addition, if the uncuffed ETT is not of a suitable size, the ETT leakage rate increases; this can create problems in reaching target tidal volumes in mechanical ventilation [4, 6]. Therefore, to reduce the risk of aspiration in laparoscopic surgery, to prevent multiple laryngoscopies and intubations, and for more accurate monitoring of tidal volume and end-tidal carbon dioxide in ventilation, a cuffed ETT is also used [1, 3]. However, the cricoid of the tracheal tubes poses a serious risk of airway damage in children due to the cuff being inserted between the subglottic region and the vocal cords and due to the fact that all pediatric cuffed tubes have the main design defects that can cause airway complications [4, 6–9].

Today, preference is given according to the advantages and disadvantages of ETT in pediatric patients; however, there are still no precise rules. In the literature, there are no reports of previous studies evaluating the use of cuffed vs. uncuffed ETT in pediatric patients under the age of 5 years in both laparotomy and laparoscopic surgeries. The aim of this investigation is to assess whether there is a relationship between the use of perioperative mechanical ventilation management and the frequency of development of complications.

## 2. Methods

Patients who underwent laparotomy (LT group) or laparoscopic (LS group) surgery between 1 and 60 months after receiving hospital ethics committee approval and who underwent uncuffed ETT during intubation were retrospectively included in the study. Patients with neuromuscular or congenital metabolic diseases of childhood, asthma and other lung diseases, intubation with a cuffed ETT, a difficult airway in a mask or intubation, postoperative intubation in the case of need, or tracheostomy were excluded from the study.

All patients received premedication with oral midazolam 0.5 mg/kg at least 30 minutes before induction of general anesthesia. Intraoperative monitoring was provided with heart rate, noninvasive blood pressure, peripheral oxygen saturation, and capnography. The induction of general anesthesia was achieved with 5-8% sevoflurane (Sevorane®; Abbott Ltd., UK), 50% oxygen, 50% air, 1 *μ*g/kg remifentanil (Rentanil®; Vem Ltd., Ankara, Turkey), and 0.45 mg/kg rocuronium bromide (Myocron®; Vem Ltd., Ankara, Turkey) in all children. The intubation was performed by the experienced anesthesiologist, who had >10 years of experience in pediatric anesthesia. The size of the uncuffed ETT (Chilecom®; Wellkang Ltd., London, UK) was formulated based on the internal diameter (ID) in mm: ID = 4.5 + (age in years/4) (Penlington's formula). Tube selection was carried out so as to start endotracheal intubation with the largest uncuffed tube and continue intubation with a smaller uncuffed tube if the tube encountered resistance and could not pass. After the position of the tube was confirmed by auscultation, capnography, and spO_2_ ≥ 96, tube detection was performed. After intubation, all patients underwent gastric decompression with a nasogastric tube followed by mechanical ventilation in pressure control ventilation (PCV) mode (Avance CS^2^®; Datex Ohmeda, Madison WI, USA).

Respiration parameters such as end-tidal carbon dioxide (etCO_2_), peak inspiratory pressure (PIP), inspiration tidal volume (TVi), and expiration tidal volume (TVe) of all preoperative and perioperative patients were measured through a flow sensor connected between the ETT and the ventilator circuit.

Mechanical ventilation (MV) settings were adjusted to PIP 10-30 cmH_2_O, TVe 8-10 ml/kg, inspiration : expiration ratio 1 : 2, and positive end-expiratory pressure (PEEP) 5 cmH_2_O. The number of inhalations was adjusted within the limits of etCO_2_ 25-40 mmHg. The value of ETT leak was calculated (%) = (TVi − TVe)/TVi × 100 [6,8]. A larger number tube size was decided by the attending anesthetist and was based on the existence of PIP > 30 cmH_2_O or TVe < 8 ml/kg or ETT leak > 20% in the MV. Pneumoperitoneum was induced by insufflating carbon dioxide (CO_2_) into the abdomen with the intra-abdominal pressure maintained at 10-11 mmHg (Electronic Endoflator; Karl-Storz, Tuttlingen, Germany), with the patient's position recorded as flank, supine, lithotomy position, or 0-25° reverse Trendelenburg tilt. After pneumoperitoneum, TVe was adjusted to 8-10 ml/kg and etCO_2_ to 25-40 mmHg, gradually increasing the values of PIP and respiration. After intraoperative PIP > 30 cmH_2_O or pneumoperitoneum and in the case of less than 8 ml/kg etCO_2_ over 40 mmHg, manual ventilation was changed from PCV mode, and etCO_2_ was reduced below 40 mmHg. In the maintenance of anesthesia, 4 l/min, 50% oxygen and air, 2%-4% sevoflurane (MAC 1-1.3), and remifentanil 0.05-0.2 *μ*g/kg/min were provided. The sexes of patients, age, body weight, height, ASA, operation, tube change, number of breaths, PIP, peripheral oxygen saturation (spO_2_), respiratory count, etCO_2_, duration of operation, additional drugs, ETT numbers, and tube changes were recorded. In addition, coughing, laryngospasm, or stridor at extubation was recorded. Demographic data, ventilation parameters (breath rate, PIP, and etCO_2_), size of ETT, the reason for tube change, aspiration, coughing, laryngospasm, or stridor was recorded.

### 2.1. Statistical Analysis

Data were analyzed by using the SPSS 23 package program for Windows. While evaluating the study data, descriptive statistics were presented as mean and standard deviation. Comparison of quantitative data between groups was done by the Mann−Whitney *U* test. Comparison of categorical variables between groups was done by the chi-squared test; continuous variables with a normal distribution were evaluated by using one-way analysis of variance, while continuous variables with a nonnormal distribution were evaluated by using Kruskal-Wallis variance analysis. Statistical significance was evaluated at the level of *p* < 0.05.

## 3. Results

A total of 102 patients, 38 females and 64 males, with a mean age of 10.9 ± 8.1 months, body weight 7.1 ± 3.7 kg, and height 67 ± 15 cm, were included (Table 1). Surgical applications included acute abdominal surgery, ectopic testicle exploration, inguinal hernia repair, pyeloplasty, renal mass, colostomy opening or closing, and acute abdominal expression. Hirschsprung pull-through, Nissen fundoplication, liver biopsy, and cholangiography were used (Table 2).

When the patients' demographic data were examined, there was no significant difference between the LT and LS groups in terms of age, gender, and ASA scores (*p* = 0.63, *p* = 0.71, and *p* = 0.09, respectively). Patients' body weight and height values were significantly different between the two groups (Table 2). There was a difference in the evaluation in terms of operational distributions between the two groups and patient positions. The supine position was higher in LT group patients, and the LS group had more flank, modified lithotomy, and reverse Trendelenburg position surgeries (Table 2). The mean duration of operations was 84 ± 36.7 minutes, while in the LT group, it was 152 ± 66.5 minutes. The difference in surgery times between the groups was statistically significant (*p* < 0.0001).

The distributions of tube numbers in all cases ranged from ID 2.5 to 5 mm in diameter. Tube exchange was performed in a total of 18 patients. In patients who underwent tube exchange, 11 patients were intubated with a smaller ETT number during laryngoscopy because the ETT did not pass through the vocal cords. Others underwent endotracheal intubation; when the MV parameters were Tve < 8 ml/kg and ETT leak > 20%, a larger uncuffed tube was used due to PIP 30 cmH_2_O pressure. There was no significant difference between the groups in the frequency of tube changes (Table 3). While there was no significant difference in respiratory count values between the LT and LS groups, PIP and etCO_2_ were significantly higher in the LS group. Mean PIP values were 16.6 ± 2.1 mmHg in the LT group and 20 ± 2.8 mmHg in the LS group (Table 3). There were a total of 4 patients in the LT group and 6 patients in the LS group who changed manual ventilation from PCV. There was no significant difference in the statistical comparison between groups of patients who underwent manual ventilation. Patients with aspiration were not found in the LT and LS groups. There was no difference in the intergroup evaluation for postoperative side effects such as cough, laryngospasm, and stridor (Table 3).

## 4. Discussion

In childhood, there is no complete compromise on the choice of tubes according to the operation. In the literature, to date, there has been no publication about the use of uncuffed tubes in both laparotomy and laparoscopic surgeries in pediatric patients. We found this topic worth presenting here because we showed that the use of an uncuffed ETT in children under 5 years of age undergoing laparotomy and laparoscopic surgeries occurs smoothly and without complications.

In general anesthesia applications, cuffed or uncuffed tubes used in pediatric patients have both advantages and disadvantages. A cuffed ETT is preferred for many reasons, such as reducing the risk of aspiration, reducing air leakage, and preventing environmental contamination [1, 9, 10]. However, the preferred cuffed ETT for intubation should be smaller than the uncuffed ETT due to the structure of the cuff. But also, the use of an uncuffed ETT requires a tube with a larger ID diameter of 0.5-1 mm [2, 9–11]. Despite the fact that the effect is smaller in older children, the use of a 3 mm tube, in comparison with a 4 mm tube, is associated with a threefold increase in respiratory resistance and increase in respiratory work and secretions. And also, failure to aspiration of these secretions leads to potential respiratory problems such as airway obstruction [11]. There is a risk of aspiration, especially in pediatric emergency abdominal surgeries, with the use of an uncuffed ETT [1]. In our study of children, in laparotomy and laparoscopic surgeries, we started with the largest possible uncuffed tube. Unless the largest tube was intubated, we used a smaller tube. Thus, we minimized leakage around the tube to reduce the risk of aspiration. We did not observe aspiration in any of our patients who used an uncuffed ETT, including emergency abdominal surgery operations performed with laparotomy or laparoscopic surgery.

Due to the fact that children have a short trachea, the cuffed tubes have a reduced safety limit of about 50%. When using a mesh ETT, the distal placement limit of the tracheal tube is in the carina, while the proximal cuffed border extends to the edge of the vocal cord [1, 4, 6, 7, 9]. In order to reduce the risk of subglottic damage caused by the cuffed tube and prevent side effects such as postextubation stridor, it is recommended that the uncuffed ETT be used in children under 8 years of age [1, 7, 9]. Although there are many formulas in the use of uncuffed ETT, it can still be quite difficult to find a suitable diameter tube, and accordingly, multiple laryngoscopes or intubations can be used to change the tube causes [1, 5]. In our study, multiple tube changes were performed due to the use of ETT, as in the literature; consequently, side effects were caused in some cases [1, 5]. Side effects such as laryngospasm, postextubation coughing, and stridor have often occurred in patients who underwent an uncuffed ETT replacement similar to the literature [2, 5].

In mechanical ventilation, intubation with an uncuffed ETT can pose difficulties in obtaining tidal volumes due to leakage around the tube. In addition, the small uncuffed ETT in very low-birth weight infants in the neonatal intensive care unit with insufficient tidal volumes in mechanical ventilation results in an extension of the duration [4, 6, 10]. If ETT leakage is 20%, the target tidal volume remains below 10% [6]. Disturbance of mechanical ventilation due to target tidal volume, lung compliance, and resistance changes due to ETT leakage is over 20% [4, 8]. It is recommended that a large-number tube be used to prevent secondary insufficient tidal volume development to increase ETT leak in pneumoperitoneum or mechanical ventilation, especially in the use of uncuffed ETT in laparoscopic surgeries [10]. We started intubation with the largest possible uncuffed ETT to ensure that the ETT leak value did not exceed the ideal mechanical ventilation values, in accordance with the literature. Further, in the event that the tube encountered resistance and did not pass, we performed intubation with a small uncuffed tube. In addition, an uncuffed tube may need to be replaced with another larger diameter tube for adequate ventilation of the airway; in which case, several attempts may sometimes be required before the appropriate size is found [3, 8]. In our study, laparotomy and laparoscopic operations in the pediatric patient group were performed with multiple laryngoscopies because of difficulty to pass the vocal cords or leak increase or less target tidal volumes. And also, an ETT tube was changed before the surgery started.

Today, laparoscopic operations are more often preferred in pediatric surgery. Anesthesia management performed during laparoscopic surgery, pathophysiological changes caused by pneumoperitoneum, and close follow-up of changes due to patient position are important. With an increase in intra-abdominal pressure, a decrease in tidal volume, an increase in airway resistance, a decrease in pulmonary compensation, and a decrease in functional residual capacity, disturbances in diaphragm movement may occur [1, 8, 12, 13]. In addition, in laparoscopic surgeries, carbon dioxide gas is absorbed into the bloodstream as a result of the absorption of pneumoperitoneum, causing hypercapnia [12–14]. Absorbed carbon dioxide increased while PIP should be increased in PCV mode in mechanical ventilation to achieve target tidal volume due to increased intra-abdominal pressure due to pneumoperitoneum. In this case, it is necessary to increase breath rates [2, 13]. The number of respirations and PIP were significantly higher due to higher carbon dioxide values in the group that underwent laparoscopic surgery; in accordance with the literature, the mechanical ventilation parameters PIP and CO_2_ were higher in patients who underwent laparoscopy, within normal physiological limits. Intraoperative increases in lung peak pressure occurred in patients undergoing both laparotomy and laparoscopic operations, but they did not reach target tidal volumes, and end-tidal carbon dioxide levels were switched from PCV mode to manual ventilation in a similar number of patients.

The choice of anesthesia technique in laparoscopic surgery depends on the patient's comorbid factors and the type of planned surgical intervention. Pneumoperitoneum and patient positions in laparoscopy cause physiopathological changes that complicate the anesthesia approach. Particularly, the Trendelenburg position restricts the diaphragm cavity depending on the weight of the intra-abdominal contents, while the diaphragm and the cephalic position of the mediastinum may cause the risk of endobronchial intubation to increase. Therefore, changing the Trendelenburg position and ETT position may cause problems in mechanical ventilation during the operation [8, 13]. In our study, although operative distributions and operational positions are different in the laparoscopic and laparotomy surgical groups, the position of the ETT is changed in both groups, or there were no problems associated with mechanical ventilation.

## 5. Summary

In conclusion, we can start with the largest possible uncuffed tube to decrease ETT leak so that both laparotomy and laparoscopic operations in children can be achieved with safe mechanical ventilation and target tidal volume. When there is difficulty to pass the vocal cords or leak increase or less target tidal volume, the ETT tube should be changed before the surgery incision.

## Figures and Tables

**Table 1:** Demographic and ventilation parameters of patients.

Parameters	All (*n* = 102)
Gender	
Male	38 (37%)
Female	64 (63%)
Age (month)	10.9 ± 8.1
Height (cm)	67 ± 15.7
Weight (kg)	7.1 ± 3.7
ASA	
ASA 1	63 (61.8%)
ASA 2	19 (18.6%)
ASA 3	20 (19.6%)
Peak inspiratory pressure (mmHg)	18 ± 3
The frequency of the number of breaths (minutes)	17.1 ± 2.2
End-tidal carbon dioxide (mmHg)	33.1 ± 4.1
The reason for tube change	18 (17.6%)
With a small tube	11 (61%)
With a large tube	7 (39%)

Data are expressed as *n* (%) or mean ± STD. ASA: American Society of Anesthesiologists.

**Table 2:** Distributions of demographic and surgery data.

Demographic and surgery data	Laparotomy group (*n* = 48)	Laparoscopy group (*n* = 54)	*p* levels
Age (month)	8.7 ± 13	12.8 ± 10	0.63
Height (cm)	63 ± 17	70 ± 13	0.017^∗^
Weight (kg)	5.8 ± 3.6	8.2 ± 3.3	0.01^∗^
Surgical position			0.001^∗^
Supine	41 (86%)	21 (39%)	
Flank (right and left) position	4 (8%)	8 (15%)	
Lithotomy and reverse Trendelenburg	3 (6%)	25 (46%)	
Distribution of operations			0.004^∗^
Acute abstract surgery	13 (27%)	10 (18%)	
Ectopic testicle expression	1 (2%)	11 (20%)	
Pyeloplasty	2 (4%)	7 (13%)	
Inguinal hernia repair	17 (36%)	0 (0%)	
Opening or closing colostomy	8 (17%)	0 (0%)	
Hirschsprung pull-through	2 (4%)	9 (17%)	
Nissen fundoplication	0 (0%)	15 (28%)	
Excision of renal mass	2 (4%)	1 (2%)	
Liver biopsy and/or cholangiography	3 (6%)	1 (2%)	

**Table 3:** Endotracheal tube data, side effects, and mechanical ventilation parameters of groups.

Parameters	Laparotomy group (*n* = 48)	Laparoscopy group (*n* = 54)	*p* levels
Endotracheal tube			0.0001^∗^
ID 2.5 mm	2 (4%)	0 (0%)	
ID 3.0 mm	6 (13%)	0 (0%)	
ID 3.5 mm	5 (10%)	4 (7%)	
ID 4.0 mm	13 (27%)	8 (15%)	
ID 4.5 mm	10 (21%)	20 (37%)	
ID 5.0 mm	12 (25%)	22 (41%)	
Tube replacement			0.24
With a small tube	5 (10%)	6 (11%)	
With a large tube	3 (6%)	4 (7%)	
Number of breaths (minutes)	16.6 ± 2.1	17.6 ± 2.3	0.25
End-tidal carbon dioxide (mmHg)	31.5 ± 3.8	34.6 ± 3.8	0.0001^∗^
Peak inspiratory pressure (mmHg)	16.6 ± 2.1	20 ± 2.8	0.0001^∗^
Mechanical ventilation			0.96
Manual ventilation	4 (8%)	6 (11%)	
Pressure control ventilation	44 (92%)	48 (89%)	
Side effect	6 (12%)	9 (17%)	0.21
Laryngospasm	1 (2%)	1 (2%)	
Postextubation cough	4 (8%)	5 (9%)	
Postextubation stridor	1 (2%)	3 (6%)	

## Data Availability

The data used to support the findings of this study are available from the corresponding author upon request.
